# Integrated Quantitative Neuro-Transcriptome Analysis of Several Brain Areas in Human Trisomy 21

**DOI:** 10.3390/genes13040628

**Published:** 2022-04-01

**Authors:** Alejandra Rodríguez-Ortiz, Julio César Montoya-Villegas, Felipe García-Vallejo, Yecid Mina-Paz

**Affiliations:** 1 SIT Consulting—Science, Innovation & Technology, Cali 76001, Colombia; alejandra.rodriguez@correounivalle.edu.co; 2 Laboratory of Molecular Biology and Pathogenesis, Department of Physiological Sciences, School of Basic Sciences, Faculty of Health, Universidad del Valle, Cali 76001, Colombia; julio.montoya@correounivalle.edu.co (J.C.M.-V.); jesus.garcia@correounivalle.edu.co (F.G.-V.); 3 Health and Movement Research Group, Faculty of Health, Universidad Santiago de Cali, Cali 76001, Colombia; 4 Faculty of Education and Sports Sciences, Escuela Nacional del Deporte, Cali 76001, Colombia

**Keywords:** transcriptomics, brain, Down syndrome, hippocampus

## Abstract

Background: Although Down syndrome (DS) is the most frequent human chromosomal disorder and it causes mainly intellectual disability, its clinical presentation is complex and variable. Objective: We aimed to analyze and compare the transcriptome disruption in several brain areas from individuals with DS and euploid controls as a new approach to consider a global systemic differential disruption of gene expression beyond chromosome 21. Methods: We used data from a DNA microarray experiment with ID GSE59630 previously deposited in the GEO DataSet of NCBI database. The array contained log2 values of 17,537 human genes expressed in several aeras of the human brain. We calculated the differential gene expression (Z-ratio) of all genes. Results: We found several differences in gene expression along the DS brain transcriptome, not only in the genes located at chromosome 21 but in other chromosomes. Moreover, we registered the lowest Z-ratio correlation between the age ranks of 16–22 weeks of gestation and 39–42 years (R^2^ = 0.06) and the highest Z-ratio correlation between the age ranks of 30–39 years and 40–42 years (R^2^ = 0.89). The analysis per brain areas showed that the hippocampus and the cerebellar cortex had the most different gene expression pattern when compared to the brain as a whole. Conclusions: Our results support the hypothesis of a systemic imbalance of brain protein homeostasis, or proteostasis network of cognitive and neuroplasticity process, as new model to explain the important effect on the neurophenotype of trisomy that occur not only in the loci of chromosome 21 but also in genes located in other chromosomes.

## 1. Introduction

Down syndrome (DS) is one of the few chromosomal abnormalities compatible with postpartum survival, with a frequency of 1 in 700 live births and 1 in 150 conceptions [1]. However, depending on sociocultural variables, there are notable epidemiological differences among several countries [2]. A remarkable fact is that the frequency of DS is much higher at conception, given that up to 75% and 50% of DS fetuses identified during the first and second trimester, respectively, are lost before term [3,4]. There is strong evidence that most of the errors that lead to the trisomic condition are generated during meiotic processes, in which approximately 90% of the cases involving a 21 chromosome from maternal origin [5,6].

DS is the most frequent human chromosomal disorder with a complex and variable clinical presentation, and it causes mainly intellectual disability (ID). Individuals with DS also frequently develop Alzheimer disease by the fourth decade [7], and the severity of their cognition impairment is highly variable [8,9]. Despite the huge volume of knowledge about DS, the detailed molecular mechanisms of it neuropathogenesis are still not fully understood [10,11].

Up to now, two different hypotheses have been proposed to explain the DS phenotype: “Amplified developmental instability” [12] and “Gene-dosage effect” [13]. According to the first hypothesis, trisomy 21 causes a generalized genetic imbalance that disrupts evolutionarily conserved developmental pathways by decreasing developmental homeostasis and precision throughout development [14,15]. On the contrary, the “gene dosage effect” hypothesis states that the phenotype is a direct result of the cumulative effect of the imbalance of the individual genes located on the triplicated chromosome 21 [16,17]. 

To determine which one of the two hypotheses may explain the etiology of DS, several gene expression studies have been performed in mouse models or in human tissues and cell lines. Some methods used include DNA microarrays, serial analysis of gene expression (SAGE), real-time RT-PCR, RNA-seq or even proteomic approaches [18,19,20,21,22,23]. Despite an important number of experiments, the results have been contrasting, probably due to tissue specificity, developmental stages, applied experimental platforms and statistical techniques used. It is also suggested that both hypotheses are not mutually exclusive and complex processes operating in the DS phenotype could probably result in both mechanisms [24,25,26]. 

Since the brain is the structure involved in cognitive and mental disability, principal traits of the DS neurophenotype, in the present study, we analyzed the differential transcriptomic profiles of several important areas of the human brain associated with learning and memory. Our results revealed the complexity of gene expression and interacting networks in the transcriptome profiles of hippocampus and some areas of the frontal lobe, occipital lobe, temporal lobe and CBC. Moreover, our approach opens a new vision of the DS as a pathology of multiple and complex genomic variables that work together to model the pathogenesis. 

## 2. Methodology

### 2.1. Data Mining

We analyzed the differential gene expression of a brain transcriptome experiment, including 17,537 human genes. For all calculations performed in this study we used the log2 transformed expression values of free access DNA microarray experiment, whose registration code in the GEO database was GSE59630 (http://www.ncbi.nlm.nih.gov/geo/query/acc.cgi?acc=GSE59630 (accessed on 1 December 2018)), which was previously deposited by Olmos et al. [27]. This microarray was selected due to its large sample size, and because it was the most complete experiment with brain tissue. The selected microarray experiment included gene expression data of 17,537 genes from 58 post-mortem brain samples of DS patients (25 from females and 33 from males) and 58 euploid samples as normal controls (25 from females and 33 from males), classified by sex, age and brain areas including: Hippocampus (HIP), cerebellar cortex (CBC), and some cerebral cortex structures corresponding to the Dorsolateral prefrontal cortex (DFC), Orbital prefrontal cortex (OFC), Ventrolateral prefrontal cortex (VFC), Medial prefrontal cortex (MFC), Primary somatosensory cortex (S1C), Inferior parietal cortex (IPC), Primary visual cortex (V1C), Superior temporal cortex (STC), Inferior temporal cortex (ITC). Nevertheless, for the present study we decided to analyze not only the brain as a whole but also OFC, MFC, HIP and CBC brain regions which are highly associated with neurophenotype of DS.

### 2.2. Differential Gene Expression Quantification

Raw intensity log2 data of each experiment which were used for the calculation of Z-score [28]. Z-scores of the protein coding genes analyzed, were calculated according to Equation (1):

(1) $$Z\text{-}\mathrm{score}=\frac{\left(Log\;intensity\;of\;G-mean\log intensity\;G\ldots Gn\right)}{Standard\;Deviation\log G\ldots Gn}$$

Equation (1), Z-score formula.

All Z-score values were normalized on a linear scale −3.0 ≤ 0 ≥ +3.0 (two-tailed *p* value < 0.001). From Z-score data, we calculated the mean values per gene and per structure in brain samples of DS and euploid controls. These data were used to calculate the Z-ratio with the equation proposed by Cheadle et al. [28] (Equation (2)) is a measurement that estimates differential gene expression. According to this equation, those genes with Z-ratio values over 1.96 are considered over-expressed [29].

(2) $$Z\text{-}\mathrm{ratio}=\frac{\left[\left(Z\text{-}{\mathrm{score}}_{G1ave}\right)DS-\left(Z\text{-}{\mathrm{score}}_{G1ave}\right)Con\right]}{SD\;of\;Z\text{-}\mathrm{score}\;differences_{G1\ldots Gn}}$$

Equation (2), Z-ratio formula.

### 2.3. Statistical Analysis

Statistical analyses for comparing mean values of Z-ratio were performed among the different brain cortex structures between DS patients and euploid controls. The Wilcoxon signed-rank test/Two-tailed was used to calculate differences between medians of two samples. The *p*-values were calculated using the web tool *p*-value from Z-score Calculator (https://www.socscistatistics.com/pvalues/normaldistribution.aspx (accessed on 1 December 2018)). In all cases we used an α 0.05 to test the significance of H_0_. To calculate the statistical differences in the mean log2 values of DS and euploid controls for sex, age, hippocampus, cerebellar and brain cortex structures, we applied the t-test for two paired samples/Two-tailed test with an α of 0.05. For the correlation analysis we used the Microsoft Excel^®^ tool for graphics design. GO categories were obtained using the free visualization platform Cytoscape 3.9.

## 3. Results

### 3.1. Differences in the Global Gene Over-Expression in Brain in Chromosomes and Structures of Down Syndrome Individuals

Overall, we found that 2.77% (486/17,537) of overexpressed analyzed coding protein genes in brains of DS individuals were differentially distributed along all human chromosomes. Chromosome 21 accounted for 14.96% (35/234) of overexpressed genes in the brain of DS samples, followed by chromosome 18 with 3.70% (10/270), chromosome 8 with 3.10% (21/677), chromosome X with 3.09% (26/842), and chromosome 12 with 2.90% (30/1034). (Table 1).

Nevertheless, every brain structure we analyzed had its own set of overexpressed genes. In some brain cortex areas, the gene overexpression values were variables and depended on the brain structure under analysis. DFC accounted for 3.43% (601/17,537) of gene overexpression; OFC the 2.7% (474/17,537); VFC 2.38% (418/17,537); ITC 2.37% (415/17,537). However, in HIP 2.43% (426/17,537) and in CBC 2.72% (477/17,537) of genes were overexpressed (Table 2).

The most associated GO-Categories biological processes to the over-expressed genes from DS samples are shown in Table 3 with their respective *p*-values (Bonferroni correction). We selected the first 10 and presented them in a decreasing order according to the *p*-value. The presence of processes associated with epigenetic such as DNA-demethylation (*p*-value 1.7208 × 10^−19^), histone deacetylation (*p*-value 3.4498 × 10^−17^), Histone H3-K4 methylation (*p*-value 6.94 × 10^−16^), and Histone H3-K9 deacetylation (*p*-value 7.87 × 10^−10^) were prominent (Table 3). 

### 3.2. Z-Ratio Correlations among Brain Structures and Age-Ranks

**Figure 1:**
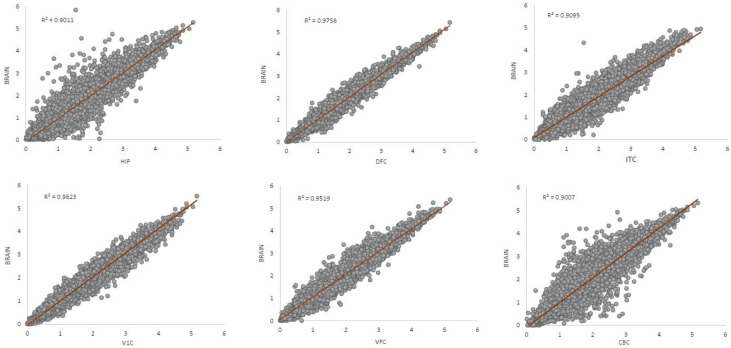
Z-ratio correlation among different brain structures and the brain as a whole. HIP. Hippocampus, DFC. Dorsofrontal Cortex, ITC. Inferior Temporal Cortex, V1C. Visual Cortex, VFC. Ventrofrontal Cortex, CBC. Cerebellar Cortex.

The correlations calculated among different DS brain structures showed that overexpression in HIP and CBC was particularly different from the one found in the brain as a whole (R^2^ = 0.9011 and R^2^ = 0.9007, respectively). DFC, on the other hand, presented the best correlation with the brain (R^2^ = 0.9756) (Figure 1).

**Figure 2:**
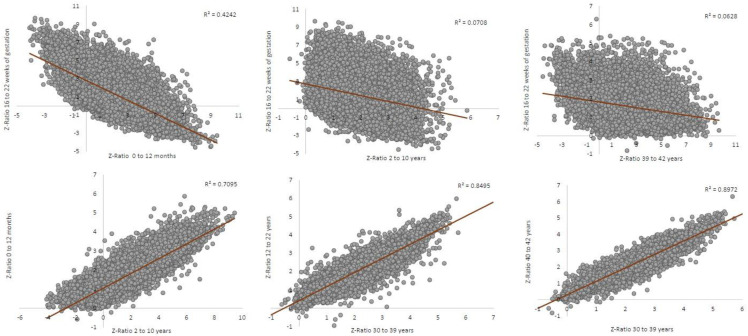
Z-ratio correlation among different age-ranks in the brain samples of Down syndrome.

Moreover, the correlations performed among different age-ranks showed a highly altered pattern dependent of the age rank that was calculated. The overexpression found for the rank of 16–22 weeks of gestation showed very low correlation coefficient with the age rank of 39–42 years (R^2^ = 0.0628), 2–10 years (R^2^ = 0.0708) and even with 0–12-month brain samples (R^2^ = 0.4242). However, the best correlation coefficient values were for 12–22 years with 30–39 years (R^2^ = 0.85) and 40–42 years with 30–39 years (R^2^ = 0.90) (Figure 2).

## 4. Discussion

The objective of this study was to analyze and compare the transcriptome of brain samples from individuals with DS and euploid controls. For that purpose, we used data from a DNA microarray experiment GSE59630, the contained log2 expression values of 17,537 human genes from postmortem brain samples of individuals with DS, and samples from euploid controls. Here, we found differences in gene expression along the whole transcriptome obtained from brain samples, not only in the genes from the chromosome 21; also, the analysis per brain areas showed that the hippocampus and the cerebellar cortex had the most different gene expression pattern when compared to the brain as a whole. 

Our findings support the hypothesis of a systemic imbalance of brain protein homeostasis, or a proteostasis network of cognitive and neuroplasticity processes as an important effect of trisomy, not only in the loci of chromosome 21, but also in genes located in other chromosomes [29,30]. It is possible that an accumulation of toxic protein aggregates caused by a failed degradative system in DS neurons negatively affects neuroplasticity processes in brain structures [31,32,33,34,35,36,37]. In this sense, our results extended the current knowledge frontier of the neurophysiological mechanisms involved in the disturbance of extensive gene expression that are remodeling the functional gene networks interaction architecture in DS brains.

One of the most important findings for this study is the global over-expression of 486 genes across the transcriptome in all chromosomes, not only in chromosome 21 nor even in the called “Down Syndrome Critical Region”, as could be expected, given that only that DS samples used had a full trisomy confirmed. There are some studies addressing this issue, mainly in murine models, however, it is important to emphasize that results in mice cannot be completely extrapolated to humans considering that human chromosome 21 is only partially analogous to mouse chromosome 16. Kahlem et al. [30] found in their study in mice that a significant fraction of genes was differentially regulated in a few tissues, suggesting additional mechanisms affecting gene expression in specific cell types. One of the possible explanations that we propose is the “cascade effect”, in which over-expressed transcription factors or epigenome regulators such as HMGN1, located in chromosome 21, affect the expression of other genes located in different chromosomes, inducing the loss of protein homeostasis in the brain. This could explain how the triplication of one of the smallest chromosomes with approximately 346 genes can cause the over-expression of 486 genes in the DS brain. In fact, Kahlem et al. [30] found that most triplicated genes coding for DNA binding proteins, including transcription factors, chromatin proteins, and RNA binding proteins, were overexpressed by a factor of about 1.5-fold. It is worth noticing that not all genes from chromosome 21 are affected. This is suggested by the dose-compensation presented and documented in trisomy 21, where we find genes that are dose-sensitive and others not sensitive.

Another interesting finding in the present study was that GO-categories, biological processes associated with the overexpressed genes, were mainly focused on epigenetic processes such as DNA methylation and histone deacetylation. Nowadays, it would be a mistake disregard the effect that epigenetics has in combination with genetics, in the development of syndromes, and DS is not the exception [32]. Genome-wide methylation studies have identified epigenetic marks in different sample tissues from individuals with DS, including skin fibroblasts, liver, placenta and brain among others [31]. 

According to our results, chromosome 21 had the highest percentage of over-expressed genes in comparison to the total of protein coding genes found in the chromosome, followed by chromosome 18, 8 and X. These results suggest that full trisomy of chromosome 21 affects not only the expression of the genes within chromosome 21, but also the expression of other genes of another different chromosomes. The dysregulation found across the transcriptome could be a “cascade effect”, initially, due to the anomalous expression of genes in chromosome 21 that regulate the expression of other genes, i.e., transcription factors. Specifically, the over-expressed genes in chromosomes 21 and 18 are involved mainly in mitochondrial processes, and, as we stated previously, one of the GO categories most associated with the overexpressed genes was ATP synthesis coupled electron transport, which takes place inside the mitochondria. Izzo et al. [38] report how mitochondrial dysfunction might affect the phenotype found in individuals with DS in aspects such as muscle hypotonia, intellectual disability and neurodegeneration, heart defects, type 2 diabetes and obesity, and immune disorders [39,40]. The study by Piccoli et al. [41] showed how in human primary lines of DS fetal fibroblasts, trisomy 21 perturbed the expression of genes involved in mitochondrial pathways, decreasing oxygen consumption and ATP content and increasing mtCa^2+^ load and ROS production. Likewise, Izzo et al. [38] in their study show how the overexpression of human genes on chromosome 21 is directly or indirectly responsible for the pathogenesis of DS phenotypic features, given that, as we stated above, many genes located in chromosome 21 can affect the expression of other genes from different chromosomes. They focused specifically on the involvement of over-expressed genes such as DYRK1A, RCAN1, NRIP1 and ATP in mitochondrial function and energy conversion, leading to mitochondrial dysfunction and chronic oxidative stress, which is consistently observed in individuals with DS [42]. 

According to our results, the expression pattern in the brain of individuals with DS during the pre-gestational period is completely different from the pattern in those who are in their late 30 to 40s, as would be expected. The brain during embryogenesis is still in formation; rearranges in synaptic connection are made throughout the brain by changes in gene expression. In contrast, when a person reaches 30–40 years, the brain is entirely formed and even though they can learn new things and new synapsis connections can be made, the expression pattern does not change drastically. This difference was visible with the negative correlation found when these two age-ranks where compared. The epigenetic here plays a crucial role; the macro and microenvironment that surrounds both age-groups are completely different, as shown in our results. 

## 5. Conclusions

Our results support the hypothesis of a systemic imbalance of brain protein homeostasis, or a proteostasis network of cognitive and neuroplasticity process as a new model to explain the important effect on the neurophenotype of trisomy that occur not only in loci of chromosome 21, but also in genes located in other chromosomes. It is likely that the sub-optimal functioning of degradative systems occurring in DS neurons in turn provide the basis for further accumulation of toxic protein aggregates, which have an indirect impact on the neuroplasticity process in several structures of the brain cortex. In this sense, our results extend the current knowledge frontier of the neurophysiological mechanisms involved in the disturbance of extensive gene expression, that are remodeling the functional gene networks interaction architecture in DS brains.

## Figures and Tables

**Table 1:** Number and percentage of coding protein genes per chromosome which are over-expressed in Down syndrome patients.

Chromosome	Protein Coding Genes/Chromosome *	Protein Coding Genes Overexpressed in DS Brain/Chromosome	Percentage **
1	2058	46	2.24
2	1309	23	1.76
3	1078	22	2.04
4	752	19	2.53
5	876	12	1.37
6	1048	29	2.77
7	989	7	0.71
8	677	21	3.10
9	786	12	1.53
10	733	20	2.73
11	1298	29	2.23
12	1034	30	2.90
13	327	9	2.75
14	830	15	1.81
15	613	7	1.14
16	873	18	2.06
17	1197	25	2.09
18	270	10	3.70
19	1472	22	1.49
20	544	14	2.57
21	234	35	14.96
22	488	6	1.23
X	842	26	3.09
Y	71	1	1.41

* Data from GRCh38.p13″. NCBI. Genome Reference Consortium. Retrieved by 8 June 2020. ** The percentage in each chromosome of DS brain, was calculated from the total of protein coding genes reported for each chromosome.

**Table 2:** Number and percentage of coding protein genes per brain structure which are over-expressed in Down syndrome patients.

Structure	Number	Percentage
Brain *	486	2.77
DFC	601	3.43
OFC	474	2.7
VFC	418	2.38
ITC	415	2.37
HIP	426	2.43
CBC	477	2.72

* Over-expressed genes based on the Z-ratio calculated with all brain regions. DFC. Dorsofrontal Cortex, OFC. Orbitofrontal Cortex, VFC. Ventrofrontal Cortex, ITC. Inferior temporal cortex, HIP. Hippocampus, CBC. Cerebellar Cortex.

**Table 3:** Top ten GO categories—biological processes involving the over-expressed genes in Down syndrome brain samples.

GO_ID	Description	*p*-Value Bonferroni
9987	DNA demethylation	1.72 × 10^−19^
43170	histone deacetylation	3.45 × 10^−17^
44260	histone H3-K4 methylation	6.94 × 10^−16^
19538	protein phosphorylation	3.03 × 10^−13^
44267	protein polyubiquitination	1.71 × 10^−12^
44237	ATP synthesis coupled electron transport	1.98 × 10^−12^
8152	5-methylcytosine catabolic process	5.44 × 10^−11^
6464	MAPK cascade	2.69 × 10^−10^
43687	post-translational protein acetylation	4.42 × 10^−10^
43412	histone H3-K9 deacetylation	7.87 × 10^−10^

## Data Availability

All data generated or analysed during this study are included in this published article. DNA microarray experiment whose registration code in the GEO database is GSE59630 (http://www.ncbi.nlm.nih.gov/geo/query/acc.cgi?acc=GSE59630 (accessed on 1 December 2018)).

## Abbreviations

SD	Down syndrome
CBC	Cerebellar cortex
DFC	Dorsolateral prefrontal cortex
HIP	Hippocampus
ID	Intellectual disability
SAGE	Serial analysis of gene expression
DNA	Deoxyribonucleic acid
RNA	ribonucleic acid
ATP	Adenosine triphosphate
ROS	Reactive Oxygen Species
OPC	Orbital prefrontal cortex
VFC	Ventrolateral prefrontal cortex
MFC	Medial prefrontal cortex
S1C	Primary somatosensory cortex
IPC	Inferior parietal cortex
V1C	Primary visual cortex
STC	Superior temporal cortex
ITC	Inferior temporal cortex
